# CSF alpha-Synuclein Seed Amplification Assay results in routine clinically collected samples

**DOI:** 10.1177/1877718X261431201

**Published:** 2026-04-07

**Authors:** Elisabeth Dinter, Julia L Margraff, Iñaki Schniewind, Nicola Reinhard, UKD SAA study group, Heinz Reichmann, Stefan Bräuer, Björn H Falkenburger

**Affiliations:** 1 Department of Neurology, Faculty of Medicine and University Hospital Carl Gustav Carus, TUD Dresden University of Technology, Dresden, Germany; 2 Department of Psychiatry and Psychotherapy, Faculty of Medicine and University Hospital Carl Gustav Carus, TUD Dresden University of Technology, Dresden, Germany; 3 German Center for Neurodegenerative Diseases (DZNE), Dresden, Germany; 4 Department of Neurology, Elblandklinikum Meißen, Meißen, Germany

**Keywords:** biological definition, Lewy body pathology, neuronal synuclein diseases, alpha-synuclein, seed amplification assay

## Abstract

**Background:**

Lewy Body pathology can be detected by alpha-synuclein-Seed Amplification Assay (αSyn-SAA) in cerebrospinal fluid (CSF) with high sensitivity and specificity in cohort studies. Yet, little is known about the results that can be expected from αSyn-SAA in CSF in samples outside cohort studies as obtained in clinical routine.

**Objective:**

This study analyzed the concordance of αSyn-SAA findings in CSF with clinical diagnosis in patients from clinical routine with diverse neurologic and psychiatric conditions.

**Methods:**

In this cross-sectional study, CSF from patients who underwent lumbar puncture for therapeutic or diagnostic purposes were tested in αSyn-SAA. Analysis included binary αSyn-SAA findings, data collected during neurological examination, and structured medical history.

**Results:**

All 356 participants (Mean Age 67.1 years, SD = 16.2; 55.9% male) were included in the primary analysis, including 90 patients with Parkinsonian syndromes, 139 with predominant cognitive disorders, 25 with other movement disorders, 35 with inflammatory or (para)neoplastic syndromes, and 67 with further diseases. αSyn-SAA was positive in all samples from patients with Parkinson's disease (41), dementia with Lewy bodies (30), pure autonomic failure (4), and in a subset of patients with Alzheimer's disease (13/46), normal pressure hydrocephalus (7/14) and others.

**Conclusions:**

αSyn-SAA findings show high concordance with a clinical diagnosis of PD and DLB. Findings are comparable to results from well-characterized cohort studies, supporting potential diagnostic value in future clinical routine. Challenges may result from the fact that αSyn-SAA detect LB co-pathology that is known from neuropathological studies for several neurodegenerative diseases.

## Introduction

Misfolded, aggregated alpha-synuclein (αSyn) is the pathological hallmark of clinically heterogeneous neurodegenerative diseases including Parkinson's disease (PD), dementia with Lewy bodies (DLB) and multiple system atrophy (MSA). In histopathologically confirmed cases of PD and DLB, aggregated αSyn is found in neuronal inclusions called Lewy bodies (LB), whereas MSA is characterized by oligodendroglial inclusions of αSyn.^1^ PD and DLB have been referred to as neuronal synuclein diseases (NSD).^2^ In all NSD, aggregates can be found in prodromal stages, before the onset of cardinal symptoms.^3^

Prion-like properties of αSyn aggregates contribute to the spread of pathology throughout the nervous system, causing motor- and non-motor symptoms. In this process, an aggregation nucleus or seed induces the conversion of unfolded α-Syn molecules towards pathological aggregates. These properties are exploited in seed amplification assays (SAA) to detect minute amounts of αSyn seeds in biomaterials such as cerebrospinal fluid (CSF).^4–6^

An early and accurate diagnosis of neurodegenerative diseases is required for life and care planning, monitoring of disease progression, and potentially for therapeutic decisions. For this reason, definitions of neurodegenerative diseases increasingly include biomarkers such as the αSyn-SAA.^2,7–9^ To date, the performance of αSyn-SAA in CSF has been studied extensively in well-characterized cohorts with PD, DLB, MSA, dementia and REM sleep behavior disorder (RBD).^3,10–12^ Yet, results from well-characterized cohorts only allow limited conclusions about the diagnostic value of a fluid biomarker in a clinical setting, because cohorts only include patients with specific diseases whereas a much broader range of diagnoses is observed in clinical routine.

In this study, we examined the results of αSyn-SAA from CSF of all patients who underwent lumbar puncture for diagnostic or therapeutic purposes in our center. αSyn-SAA was performed blinded to the patients diagnosis using an assay protocol that has been validated against a variety of other laboratories.^5,13^ This assay detects LB-associated seeding of αSyn, whereas others detect αSyn seeding associated with LB pathology as well as MSA related glial cytoplasmic inclusion pathology.^11^ The analysis of clinical data reveals a perspective on the potential diagnostic value of αSyn-SAA in clinical routine.

## Methods

### Study design

The study was conducted at the Department of Neurology, Faculty of Medicine and University Hospital Carl Gustav Carus, TUD Dresden University of Technology, Dresden, Germany. This was a blinded, monocentric, cross-sectional study conducted from October 2021 through October 2024 to evaluate the detection of LB pathology by αSyn-SAA in patients from clinical routine diagnosed with different neurologic and psychiatric diseases.

### Standard protocol approvals and patient consents

All participants provided written informed consent prior to enrollment. The study procedure was approved by the local ethics committee at TU Dresden (BO-EK-444092021, 15.10.2021) and was conducted according to the Declaration of Helsinki. All information analyzed was deidentified.

### Examination and clinical routine

The study participants were consecutive inpatient and outpatient patients who underwent lumbar puncture within the clinical routine for diagnostic or therapeutic purposes outside of emergency care. All patients underwent clinical diagnostic and therapeutic routines with comprehensive evaluation according to established guidelines by the German Neurological Society (DGN) in their current versions (www.dgn.org/leitlinie). The guidelines of the DGN follow international diagnostic criteria, including international consensus criteria for DLB, MDS criteria for PD, MSA and PSP, Gold Coast diagnostic criteria for ALS.^7,14–18^ Patients with suspected movement disorders were evaluated by movement disorder specialists, patients presenting with a predominant cognitive disorder were evaluated by an interdisciplinary team of psychiatric, neurologic and neuropsychologic specialists in the memory clinic following the German guidelines considering the DSM-5.^19^ All other patients were evaluated by specialists for neurology.

### CSF sampling

Lumbar puncture was carried out according to published guidelines.^20^ CSF was collected in polypropylene tubes and centrifuged within 90 min at 4°C and 2000 g for 10 min to remove cells and debris, then stored in aliquots at −80°C until analysis. Beta-Amyloid Ratio (1-42/1-40), levels of phosphorylated tau (pTau 181) and neurofilament light chain in CSF were measured using the Fujirebio Lumipulse system. Samples were separated from any clinical data using random number assignment.

### asynuclein monomer production

The αSyn monomer was generated and purified as previously described.^5^ In brief, BL21 (DE3) *E. coli* bacteria (Thermo Fischer Scientific) were transformed with the vector plasmid containing the human Wildtype αSyn with N-terminal His-tag. The expression was induced via autoinduction medium, cells were harvested after 18 h. The cell pellet was lysed using osmotic shock buffer [400 g/L sucrose (Carl Roth), 30 mM TRIS (Carl-Roth) pH 7.2, 2 mM EDTA (Thermo Fischer Scientific)]. The lysate was centrifuged and the pellet resolved in water. After subsequent centrifugation at 9000 g, supernatant's pH was reduced to 3.5. The solution was again centrifuged at 9000 g and the supernatant's pH increased to 7.5. Afterwards, immobilized metal ion affinity chromatography using the NGC chromatography system (BioRad) with a HisTrap FF column (Cytivia) was performed. The selected fractions were pooled and loaded on a HiTrap Q-HP anion exchange column (Cytivia); the selected fractions were subsequently dialyzed against water using a 3.5 kDa MWCO dialysis membrane (Thermo Fisher Scientific) overnight at 4°C. The concentration of the protein was measured using a NanoDrop-spectrometer (Thermo Fischer Scientific). Using this protocol, the average yield from 1 L of bacteria culture was on the order of 45–60 mg αSyn. The samples were aliquoted in 0.65–0.7 mg portions and stored at −80°C until further use.

### asynuclein seed amplification assay protocol

αSyn-SAA analyses were performed blinded to clinical data and diagnosis and conducted as previously described.^5^ The protocol was validated against the reference laboratory in Bologna and three other laboratories in a round robin trial.^5,13^

Briefly, reactions were performed in black 96-well plates with a clear bottom (Nalgene Nunc International). Each well was preloaded with six silica beads (0.8 mm in diameter, OPS Diagnostics). 15 µl of cerebrospinal fluid (CSF) were added into 85 µl reaction mix, resulting in final concentrations of 40 mM phosphate buffer, 170 mM NaCl, 0.1 mg/mL monomeric αSyn, 10 μM thioflavin T (ThT) and 0.0015% SDS per well. Every CSF was seeded in four wells (four replicates). Monomeric αSyn was filtered through a 100 kD MWCO filter immediately prior to use. Plates were sealed with plate sealer film (Nalgene Nunc International) and incubated at 42°C in a BMG FLUOstar Omega plate reader, subjected to cycles of 1 min shaking (400 rpm double orbital) and 1 min rest for at least 48 h. ThT fluorescence measurements (450 ± 10 nm excitation and 480 ± 10 nm emission; bottom read) were taken every 45 min.

Each plate included at least two negative and two positive controls, each with four replicates. Relative fluorescence units (RFU) for every time point were expressed as a percentage of the maximum intensity reached on that plate. A replicate was considered positive if fluorescence crossed a threshold within 40 h. The fluorescence threshold was defined as the average intensity of previously measured negative controls during the first 10 h of recording, plus 40 standard deviations (equaling 21% of the maximum reached on a plate). Every sample was measured in four technical replicates. A sample was considered positive if at least two replicates were positive and negative if no replicate was positive. 26 samples showed one positive replicate in the first run. Following established protocols,^5^ those samples were considered inconclusive and SAA were rerun. No sample was run more than three times to obtain a conclusive result.

### Data review and blinding

The retrospective analysis of clinical data considered the final clinical diagnosis by the physician in charge at the time of hospital discharge. Concomitant or secondary diagnoses were not included in the first analysis. Additional information collected from the patient records included CSF parameters, written reports of diagnostic imaging and symptoms collected from neurological examination and structured medical history. All information analyzed was deidentified.

Data were entered into a local electronic database, blinded to the αSyn-SAA result.

### Outcomes

The primary outcome of this study was to identify the rate of positive αSyn-SAA (using a binary outcome of seeding vs no seeding) in a clinical real-world setting. Prespecified secondary outcomes included quantitative information of the αSyn-SAA and association between αSyn-SAA positivity and CSF parameters or reported clinical features.

### Statistical analyses

Categorical variables were described as the number of patients with that feature. Continuous variables were described as mean with standard deviation or median with range. Significance in contingency tables was calculated by Fisher's exact test. *P* < .05 was considered significant. Missing data was not imputed, the number of available data is noted in each table. Data were analyzed using Microsoft Excel version 16.54 and GraphPadPrism version 10.

## Results

### Study participants

**Figure 1.**
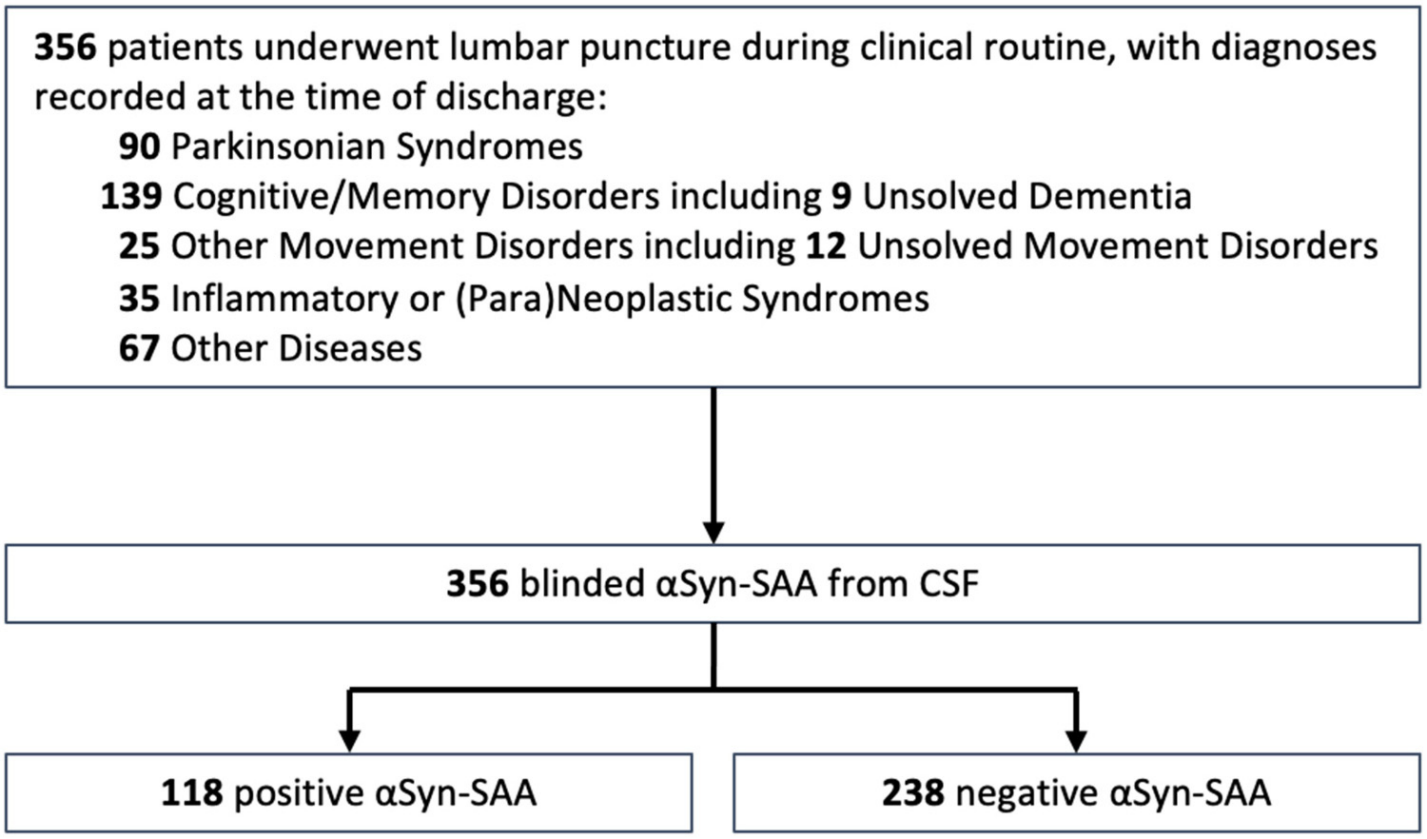
Study flow diagram. *Abbreviations:* αSyn-SAA, alpha-Synuclein Seed Amplification Assay.

From October 2021 through October 2024, 356 patients (mean [SD] age, 67.1 [16.2] years; 199 [55.9%] male) undergoing lumbar puncture within the clinical routine for diagnostic or therapeutic purposes in an academic setting consented to participate in this study. A clinical diagnosis was reached in 335 patients (94.1%). 21 patients were discharged with the diagnosis “unsolved movement disorder” or “unsolved dementia”. All diagnoses were classified into the following five groups: “Memory/Cognitive Disorders” (n = 139), “Parkinsonian Syndromes” (n = 90), “Other Movement Disorders” (n = 25), “Inflammatory/(Para)Neoplastic Syndromes” (n = 35) and “Other Diseases” (n = 67). αSyn-SAA was performed blinded to clinical diagnosis. Overall, 33.1% (118/356) of CSF samples were positive in the αSyn-SAA (Figure 1). Diagnostic groups and patients’ characteristics are summarized in Table 1, detailed information is included in Suppl. Table 1.

**Table 1.** Demographic information on disease groups.

Disease group	No. (% of total)	No. male (% of group)	Age, median (range), y	Disease duration, median (range), y
Parkinsonian Syndromes	90 (25.3)	51 (56.7)	68.5 (36–87)	3.5 (1–15)
Memory/Cognitive Disorders	139 (39.1)	89 (64.0)	78 (55–96)	2 (1–23)
Other Movement Disorders	25 (7.0)	14 (56.0)	63 (35–86)	4 (1–16)
Inflammatory/ (Para)Neoplastic Syndromes	35 (9.8)	12 (34.3)	55 (25–92)	0.5 (<0.5–11)
Other Diseases	67 (18.8)	33 (49.3)	58 (18–92)	1 (<0.5–47)
**Total cohort**	**356** **(** **100)**	**199** **(** **55.9)**	**70.5** **(** **18–96)**	**2** **(** **<0.5–47)**

A total of 356 patients who received a lumbar puncture during clinical routine were included in the study. Final diagnoses by the physician at discharge were organized into five groups: Parkinsonian syndromes, memory/cognitive disorders, other movement disorders, inflammatory/(para)neoplastic syndromes and other diseases.

*Abbreviations:* No., total Number; y, years.

### Results of CSF αSyn-SAA in Parkinsonian syndromes

**Figure 2.**
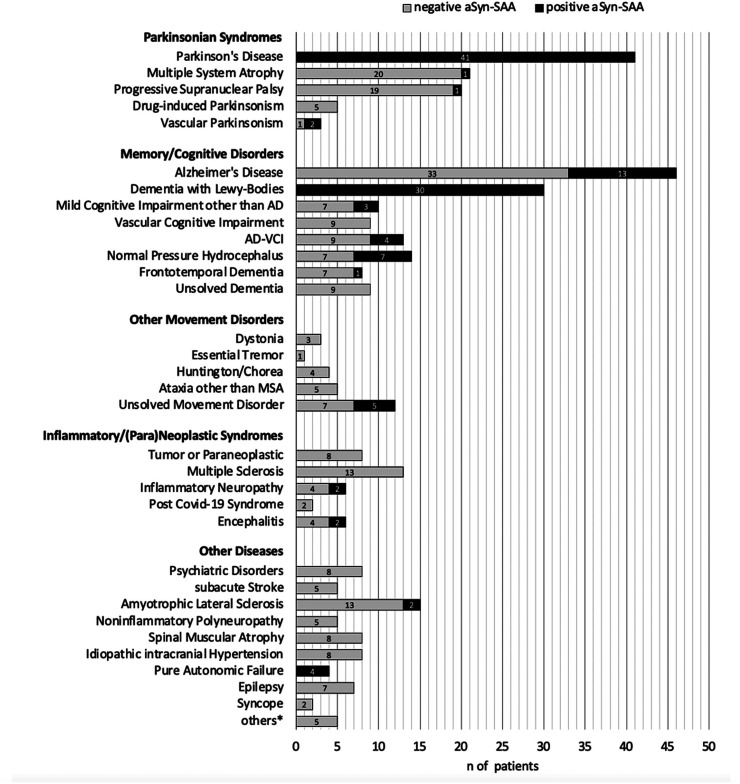
Clinical diagnosis and αSyn-SAA result. Depicted diagnoses were made by the physician in charge based on current clinical guidelines. Digits in bars depict total number of patients showing negative (grey bar) and positive (black bar) αSyn-SAA results. *Migraine, Myasthenia Gravis, Leukodystrophy, Mitochondriopathy, Traumatic Encephalopathy.

*Abbreviations:* AD-VCI, mixed Alzheimer's Disease and Vascular Cognitive Impairment; αSyn-SAA, alpha-Synuclein Seed Amplification Assay; MSA, Multiple System Atrophy; n, total number of patients.

In patients clinically diagnosed with PD, the αSyn-SAA was positive in all 41 samples (100% positive) (Figure 2). In patients with MSA, the αSyn-SAA was positive in 1 of 21 patients (4.8% positive). Note that a negative result is expected in the assay variant used here.^4,21^ In patients with progressive supranuclear palsy, the αSyn-SAA was positive in 1 of 20 samples (5% positive). CSF from all five patients with a clinical diagnosis of drug-induced parkinsonism was negative (0% positive). 2 out of 3 patients with vascular parkinsonism showed positive αSyn-SAA results (66.7% positive); note that in these patients, vascular parkinsonism was suspected due to strategic vascular lesions and an absent levodopa response.

### Results of CSF αSyn-SAA in memory/cognitive disorders

CSF from all 31 patients with a clinical diagnosis of DLB was positive in the αSyn-SAA (100% positive) (Figure 2). In CSF from patients with Alzheimer's disease (AD), αSyn-SAA was positive in 13 of 46 cases (28,3% positive). All 9 samples of patients with vascular cognitive impairment (VCI) showed negative results (0% positive), 4 positive results were obtained in 9 patients with a mixed form of AD and VCI (AD-VCI) (30.8% positive). CSF of patients with idiopathic normal pressure hydrocephalus (iNPH) was positive in 7 out of 14 cases (50% positive). This is in line with recent findings of LB pathology in NPH in vivo.^22–24^ Among 8 patients with clinical diagnosis of frontotemporal dementia (FTD), including non-fluent Primary Progressive Aphasia and Behavioral Variant FTD, CSF was positive in 1 patient (12.5% positive). In CSF from 10 patients with mild cognitive impairment other than AD or VCI, αSyn-SAA was positive in 3 cases (30% positive). CSF from all 8 patients with unsolved dementia was negative (0% positive); the differential diagnoses included suspected non-Alzheimer's pathology and limbic-predominant age-related TDP-43 encephalopathy.

### Results of CSF αSyn-SAA in other movement disorders

None of the CSF samples from patients with a clinical diagnosis of other movement disorders such as ataxia other than MSA (n = 5), essential tremor (n = 1), dystonia (n = 3), Huntington's disease and unspecified chorea (n = 4) were positive (0% positive) (Figure 2). The αSyn-SAA was positive in CSF from 5 of 12 patients with unsolved diagnoses of gait or movement disorders (41.7% positive); suspected differential diagnoses are listed in Suppl. Table 2.

### Results of CSF αSyn-SAA in inflammatory/(para)neoplastic diseases

Most samples from patients with clinically diagnosed inflammatory or (para)neoplastic syndromes showed negative results in αSyn-SAA (Figure 2). In particular: 13 cases of multiple sclerosis, 2 patients with post COVID-19 syndrome and 8 cases of cancer-related syndromes remained negative in αSyn-SAA. Interestingly, αSyn-SAA was positive in 2 of 6 cases with encephalitis (33.3% positive). Among the 6 patients with inflammatory neuropathies, including chronic and acute inflammatory demyelinating polyneuropathies, αSyn-SAA was positive in 2 cases, both with Guillan-Barré syndrome (33.3% positive).

### Results of CSF αSyn-SAA in other diseases

αSyn-SAA was positive in 2 of 15 cases with a clinical diagnosis of amyotrophic lateral sclerosis (ALS) (13,3% positive) (Figure 2). The αSyn-SAA was also positive in all 4 cases with pure autonomic failure (PAF) (100% positive). This is in line with the LB pathology known from neuropathological studies.^25^ αSyn-SAA was negative in all 48 cases with other diagnoses (0% positive), including psychiatric diagnoses (e.g., schizophrenia and dissociative disorders), subacute stroke, non-inflammatory polyneuropathy, spinal muscular atrophy, idiopathic intracranial hypertension, epilepsy, cardiac syncope and others (Figure 2).

### Quantitative information of CSF αSyn-SAA

The number of positive replicates in αSyn-SAA is discussed to contain additional information, e.g., on the load of aggregates or the disease stage.^5,26^ In CSF from patients with DLB, 87% of cases showed 4/4 positive replicates, whereas, in PD, only 54% of the cases showed 4/4 positive replicates (Table 2). This difference between DLB and PD may be explained by the fact that DLB patients have a higher load of LB in neuropathological analyses.^27^ The median clinical disease duration was two years for DLB and four years for PD and thus cannot explain this discrepancy (Suppl. Table 1). In AD, only 46% of positive cases showed 4/4 positive replicates. Considering αSyn-SAA positivity reflecting LB co-pathology in vivo, these observations may indicate that the number of positive replicates reflects the burden of LB co-pathology in patients with AD, consistent with recent findings from neuropathological studies validating SAA performance.^28^

**Table 2.** Quantitative information of αSyn-SAA result.

Clinical diagnosis	No.	αSyn-SAA result			
		Positive Replicates			
		0/4	2/4	3/4	4/4
**Parkinsonian Syndromes**					
Parkinson's Disease	41	0	7	12	22
Multiple System Atrophy	21	20	1	0	0
Progressive Supranuclear Palsy	20	19	0	0	1
Drug-induced Parkinsonism	5	5	0	0	0
Vascular Parkinsonism	3	1	0	1	1
**Memory/Cognitive Disorders**					
Alzheimer's Disease (AD)	46	33	5	2	6
Dementia with Lewy-Bodies	30	0	1	2	27
Mild Cognitive Impairment other than AD	10	7	1	1	1
Vascular Cognitive Impairment (VCI)	9	9	0	0	0
AD-VCI	13	9	2	0	2
Normal Pressure Hydrocephalus	14	7	1	3	3
Frontotemporal Dementia	8	7	0	0	1
Unsolved Dementia	9	9	0	0	0
**Other Movement Disorders**					
Dystonia	3	3	0	0	0
Essential Tremor	1	1	0	0	0
Huntington/Chorea	4	4	0	0	0
Ataxia other than MSA	5	5	0	0	0
Unsolved Movement Disorder	12	7	2	1	2
**Inflammatory/(Para)Neoplastic Syndromes**					
Tumor or Paraneoplastic	8	8	0	0	0
Encephalitis	6	4	0	1	1
Post Covid-19 Syndrome	2	2	0	0	0
Inflammatory Neuropathy	6	4	1	1	0
Multiple Sclerosis	13	13	0	0	0
**Other Diseases**					
Psychiatric Disorders	8	8	0	0	0
subacute Stroke	5	5	0	0	0
Amyotrophic Lateral Sclerosis	15	13	0	1	1
Noninflammatory Polyneuropathy	5	5	0	0	0
Spinal Muscular Atrophy	8	8	0	0	0
Idiopathic Intracranial Hypertension	8	8	0	0	0
Pure Autonomic Failure	4	0	1	1	2
Epilepsy	7	7	0	0	0
Syncope	2	2	0	0	0
others*	5	5	0	0	0

*Migraine, Myasthenia Gravis, Leukodystrophy, Mitochondriopathy, Traumatic Encephalopathy.

*Abbreviations:* αSyn-SAA, alpha-Synuclein Seed Amplification Assay; No., absolute number of patients.

### Results of αSyn-SAA, secondary diagnosis and CSF parameters

No association between αSyn-SAA positivity and documented secondary diagnoses was observed (Suppl. Table 3), including stroke, diabetes, and autoimmune diseases. Interestingly, current or past tobacco use was documented in fewer patients with a positive αSyn-SAA result (p < .04 from Fisher's exact Test). No systematic associations were detected between αSyn-SAA positivity and the number of cells in CSF, elevated total protein concentration, blood-brain-barrier disturbance, oligoclonal bands or Alzheimer's pathology in CSF (Suppl. Table 4).

### αSyn-SAA results in context of reported symptoms

In clinical practice, αSyn-SAA would be used in patients with specific symptoms but unknown diagnoses. To simulate the application of αSyn-SAA in patients with Parkinsonian symptoms, the subset of 121 patients with akinetic-rigid syndromes were plotted with the clinical diagnosis (Table 3a). In this subset, 83% of patients with positive aSyn-SAA had a clinical diagnosis of PD or DLB, and none of the patients with a negative αSyn-SAA had a diagnosis of PD or DLB; unknown dementia and unknown movement disorders were not included in this analysis.

**Table 3.** Results of αSyn-SAA in patients with selected symptoms.

a) akinetic-rigid syndrome		
Clinical diagnosis	Pos. SAA No.	Neg. SAA No.
PD	41	0
DLB	18	0
AD-VCI	2	0
MCI	1	0
AD	4	3
VP	2	1
iNPH	2	1
UMD	1	5
PSP	1	18
Encephalitis	0	1
UD	0	1
FTD	0	3
VCI	0	3
DIP	0	4
MSA	0	15
Sum*	**71 (117)**	**50 (235)**
b) predominant cognitive impairment		
DLB	30	0
iNPH	5	5
AD	13	32
AD-VCI	4	9
MCI	3	7
FTD	1	5
MS	0	1
Stroke	0	1
VCI	0	9
UD	0	9
PSP	0	14
Sum*	**56 (118)**	**92 (258)**

* number of patients with selected symptom documented as present at timepoint of CSF sampling (number of patients with available data).

a) In 121 of 352 patients akinetic-rigid syndrome was documented as present at timepoint of lumbar puncture. Depicted clinical diagnoses were given by the physician in charge.

b) 148 of 356 patients were considered suffering from primary cognitive impairment as they underwent diagnostic procedures in memory clinic. Depicted clinical diagnoses were given by the physician in charge.

*Abbreviations:* AD, Alzheimer's Disease; DIP, drug-induced Parkinsonism; DLB, dementia with Lewy-Bodies; FTD, Frontotemporal Dementia; iNPH, idiopathic Normal Pressure Hydrocephalus; MCI, Mild Cognitive Impairment other than AD; MS, Multiple sclerosis; MSA, Multiple System Atrophy, Neg., negative; No., Total number of patients; PD, Parkinson's Disease; Pos., positive; PSP, Progressive Supranuclear Palsy; SAA, alpha-Synuclein Seed Amplification Assay; UD, Unsolved Dementia; UMD, Unsolved Movement Disorder; VCI, Vascular Cognitive Impairment; VP, Vascular Parkinsonism.

Similarly, we plotted the αSyn-SAA results of all 148 patients with predominant cognitive impairment, using their visit to the memory clinic as proxy for this chief complaint (Table 3b). In this subset, 57% of patients with a positive αSyn-SAA had a clinical diagnosis of DLB, and none of the patients with a negative αSyn-SAA had a diagnosis of DLB. Again, patients with dementia of unknown origin were not included in the analysis.

An overview of αSyn-SAA results in patients with hallucinations, suspected RBD and other sleeping disorders, autonomous dysfunction, hyposmia and depression are included as supplemental material (Suppl. Table 5c-j).

## Discussion

This blinded, cross-sectional study confirmed a high concordance of αSyn-SAA results in CSF with a clinical diagnosis of PD or DLB. This finding from patients in clinical routine is consistent with several cohort studies.^10,26,28–30^

All patients with a clinical diagnosis of PD or DLB showed a positive αSyn-SAA result. The median disease duration in our population was 4 years for PD and 2 years for DLB, which supports the certainty of the clinical diagnoses (Suppl. Table 1). Positive αSyn-SAA have been documented even in prodromal PD, suggesting that the agreement between clinical diagnosis and αSyn-SAA result would be similar when patients with shorter disease duration are examined.^3,29^ The proportion of positive technical replicates was higher in DLB than in PD (Table 2), supporting the notion that the αSyn-SAA may contain quantitative information about symptom severity or disease progression and consistent with previous findings.^5,31,32^ Yet, this needs to be confirmed in larger cohorts.

We did not observe negative αSyn-SAA results in patients with a clinical diagnosis of PD, DLB or PFA, i.e., for diagnosis with known LB pathology. Yet, we did detect positive αSyn-SAA results in patients with several other diseases. Specifically, we observed 5–13% positive αSyn-SAA in patients with ALS, FTD and PSP, which compares to 5–15% of patients with LB pathology in neuropathological cohorts.^33,34^ Among the patients with AD, 28% showed a positive αSyn-SAA, which is consistent with recent observations of SAA analysis in large AD cohorts.^35,36^

The positive αSyn-SAA in patients with inflammatory diseases in this cohort is interesting and in line with the hypothesis form patient data and translational models that inflammatory environments might trigger or promote αSyn pathology in PD.^37–40^ At this point, we cannot discriminate whether the positive αSyn-SAA resulted from seeds of aggregated αSyn, which could be transiently present in inflammatory stages, or reflected incidental LB pathology. The fact that no positive αSyn-SAA was detected in CSF from patients with multiple sclerosis, and that the CSF WBC count had no association with positive αSyn-SAA (Suppl. Table 4), suggests that an “inflammatory milieu” alone is not sufficient for a positive αSyn-SAA.

Overall, the rates of positive αSyn-SAA we observed in our clinical sample are similar to the rate of LB pathology in neuropathological and SAA cohort studies. This supports the conclusion that αSyn-SAA report LB pathology. As a consequence, we assume that many of the positive αSyn-SAA results in diseases other than PD or DLB reflect LB co-pathology. Detection of co-pathology in neurodegenerative diseases might become therapeutically relevant; patients with positive αSyn-SAA and pathological amyloid biomarkers are of particular interest. Indeed, amyloid biomarkers are pathological in 25% of patients with DLB and in 3% of patients with PD.^41,42^ Conversely, LB-copathology is described in 20–30% of AD cases.^36^ In our sample, there was no statistically significant association between these two pathologies (Suppl. Table 4). In neuropathological and biomarker series, the occurrence of multiple pathologies was associated with faster disease progression in clinical DLB, PD and AD.^12,35,41–44^ Therefore, the possibility to detect LB co-pathology using biomarkers is relevant for patient counseling and prognostic evaluations. In addition, biomarkers for co-pathologies are likely to influence biomarker-based criteria for diagnoses in early disease stages and for recruitment in preclinical trials. We also anticipate that co-pathologies will be shown to affect the response to disease-modifying treatments, e.g., with anti-amyloid therapies in patients with AD.

Because positive αSyn-SAA and LB co-pathology are common in several neurodegenerative diseases, the standard αSyn-SAA is unlikely to replace clinical assessments for making a diagnosis. Still, the high concordance between αSyn-SAA findings and the clinical diagnosis observed here confirms that the αSyn-SAA would be helpful for reaching a diagnosis in clinical routine. In the context of unsolved movement disorders, a positive SAA result could possibly confirm LB-pathology suspected based on the clinical presentation. Yet, a positive SAA result should not justify a diagnosis of PD without considering the clinical presentation.

This study has several limitations. As any real-world study, the cohort is heterogeneous, the number of patients for some individual diagnoses is small, and the patients are not characterized as well as in dedicated cohorts. The gold standard for diagnosis was a comprehensive evaluation according to established guidelines in an academic setting, but not confirmed by neuropathology. Additional biomarkers such as PET for amyloid or tau were not performed; PET, dopamine transporter SPECT and genetic testing were not available in all patients (Suppl. Table 1). RBD was diagnosed based only on anamnestic information; hyposmia and orthostatic hypotension were not confirmed by diagnostic tests in every case. Patients were included at a single site and systematic clinical follow-up has not been conducted yet. On the other hand, the information that was available in this study is representative for the clinical context in which the αSyn-SAA might be interpreted in the future emphasizing the challenges faced for diagnosing neurodegenerative diseases in routine care.

## Conclusion

Our data suggests a high diagnostic value of αSyn-SAA in future clinical routine, based on high concordance with expected LB pathology in a broad spectrum of neurologic conditions. Positive αSyn-SAA results were observed in all patients with PD and DLB. Most patients with other diseases showed negative αSyn-SAA results, except for some neurodegenerative for which LB co-pathology is known from neuropathological series. Consequently, we do not expect that the αSyn-SAA will replace a clinical diagnosis of PD or DLB. Rather, clinicians will be required to interpret αSyn-SAA results in concert with clinical signs and symptoms. For this task, knowing the prevalence of positive and negative αSyn-SAA results in different diseases will be important.

## Supplemental Material

sj-docx-1-pkn-10.1177_1877718X261431201 - Supplemental material for CSF alpha-Synuclein Seed Amplification Assay results in routine clinically collected samples Supplemental material, sj-docx-1-pkn-10.1177_1877718X261431201 for CSF alpha-Synuclein Seed Amplification Assay results in routine clinically collected samples by Elisabeth Dinter, Julia L Margraff, Iñaki Schniewind, Nicola Reinhard, UKD SAA study group, Heinz Reichmann, Stefan Bräuer and Björn H Falkenburger in Journal of Parkinson's Disease
