# Supplementary material for: CSF alpha-Synuclein Seed Amplification Assay results in routine clinically collected samples
Source: J Parkinsons Dis. 2026 Apr 7:1877718X261431201. Online ahead of print. doi: 10.1177/1877718X261431201 (PMC13447608; doi:10.1177/1877718X261431201)
Supplement: Supplementary file 1 [file sj-docx-1-pkn-10.1177_1877718X261431201.docx]

**Supplemental Table 1:** Demographic data, clinical scores, imaging and CSF parameters

| **Clinical diagnosis** | **No.** | **Demography** | | | **Imaging** | | | **CSF parameter** | | | |
| --- | --- | --- | --- | --- | --- | --- | --- | --- | --- | --- | --- |
|  |  | **No. male (%)** | **Age at sampling^a^** | **Disease duration^a^** | **MRI/CT NPH+^b^** | **MRI**  **CAA+^c^** | **SPECT/ F-Dopa-PET +^d^** | **Cell count^e^** | **Protein concentration^f^** | **AD-pathology^g^** | **NfL >90^th^ perc ^h^** |
| **Parkinsonian**  **Syndromes** |  |  |  |  |  |  |  |  |  |  |  |
| PD | 41 | 30 (73.2) | 64 (36-87) | 4 (1-15) | 4/42 | 0/42 | 15/15 | 1.5±0.95 | 509.5±333.8 | 2/29 | 6/33 |
| MSA | 21 | 9 (42.9) | 66 (51-84) | 4 (1-7) | 0/21 | 0/18 | 6/6 | 1.2±0.5 | 491.7±202.8 | 0/15 | 18/20 |
| PSP | 20 | 8 (40) | 75 (59-84) | 2 (1-6) | 0/20 | 0/20 | 8/8 | 1.7±0.8 | 434.3±136.2 | 2/19 | 17/19 |
| DIP | 5 | 1 (20) | 69 (55-72) | 8 (2-15) | 0/5 | 0/5 | NA | 1.8±1.1 | 375.0±142.5 | 0/5 | 0/4 |
| VP | 3 | 3 (100) | 67 (55-69) | 1 (1-7) | 0/3 | 0/3 | 0/1 | 1.5±0.7 | 525.5±43.1 | 0/2 | 2/2 |
| **Memory/Cognitive Disorders** |  |  |  |  |  |  |  |  |  |  |  |
| AD | 46 | 29 (63) | 79.5 (57-92) | 2.5 (1-10) | 1/46 | 3/46 | 0/1 | 1.6±1.0 | 547.0±176.2 | 45/45 | 12/42 |
| DLB | 30 | 21 (70) | 79 (60-90) | 2 (1-11) | 0/30 | 0/30 | 4/4 | 1.3±0.7 | 515.4±182.4 | 15/30 | 8/30 |
| MCI^i^ | 10 | 5 (50) | 69 (56-82) | 3 (1-6) | 0/10 | 1/10 | NA | 1.5±0.9 | 552.0±197.9 | 0/10 | 2/10 |
| VCI | 9 | 5 (55.6) | 79 (75-85) | 3 (1-7) | 1/9 | 1/9 | NA | 2.1±1.5 | 556.6±176.0 | 0/9 | 4/9 |
| AD-VCI | 13 | 9 (69.2) | 81 (69-96) | 1 (1-7) | 3/13 | 2/12 | NA | 2.2±1.3 | 629.2±234.3 | 12/13 | 7/12 |
| iNPH | 15 | 12 (85.7) | 75 (55-90) | 1.5 (1-10) | 14/14 | 0/14 | 1/1 | 2.5±4.5 | 536.7±158.4 | 3/13 | 4/13 |
| FTD | 8 | 4 (50) | 69 (62-80) | 3 (1-5) | 0/8 | 0/8 | 1/1 | 1.1±0.4 | 465.5±145.8 | 0/8 | 7/8 |
| UD | 9 | 4 (44.4) | 79 (63-90) | 1 (1-4) | 0/9 | 0/9 | NA | 1.8±1.1 | 584.0±162.2 | 0/9 | 4/9 |
| **Other Movement Disorders** |  |  |  |  |  |  |  |  |  |  |  |
| Dystonia | 3 | 1 (33.3) | 57 (44-66) | 4 (2-11) | 0/2 | 0/2 | 1/0 | 1.3±0.6 | 592.3±291.7 | 0/2 | 0/3 |
| ET | 1 | 1 (100) | 72 | 4 | 0/1 | NA | 0/1 | 1 | 450 | 1/1 | NA |
| HD/Chorea | 4 | 3 (75) | 66 (39-70) | 5 (1-11) | 0/4 | 0/4 | NA | 1.0±0 | 501.5±226.9 | 1/2 | 0/2 |
| Ataxia^k^ | 5 | 3 (60) | 46 (35-60) | 6 (4-16) | 0/4 | 0/4 | NA | 5.5±6.4 | 415.5±110.8 | 0/2 | 0/4 |
| UMD | 12 | 6 (50) | 75 (45-86) | 4 (1-15) | 3/12 | 0/11 | 2/3 | 1.3±0.5 | 433.3±126.7 | 2/11 | 5/11 |
| **Inflammatory/**  **(Para)Neoplastic** |  |  |  |  |  |  |  |  |  |  |  |
| Tumor or Paraneoplastic | 8 | 4 (50) | 63 (54-71) | 2.5 (0.5-6) | 0/8 | 0/8 | NA | 9.8±13.4 | 749.3±525.0 | 0/1 | 3/5 |
| Encephalitis | 6 | 5 (83.3) | 68 (25-85) | 0.5 (<0.5-4) | 0/6 | 1/6 | 0/1 | 24.2±40.3 | 628.8±107.9 | 1/4 | 4/5 |
| Post Covid-19 Syndrome | 2 | 0 (0) | 43 (32-53) | 0.5 (0.5) | 0/1 | 0/1 | NA | 2.0±1.4 | 233.5±62.9 | NA | 0/1 |
| Inflammatory Neuropathy | 6 | 1 (16.7) | 64 (29-92) | 0.8 (0.5-11) | 0/5 | 0/5 | NA | 3.8±2.7 | 705.7±453.9 | 1/2 | 1/4 |
| MS | 13 | 2 (15.4) | 35 (26-77) | 0.5 (<0.5-9) | 0/13 | NA | NA | 15.3±14.9 | 409.4±131.3 | 0/2 | 5/8 |
| **Other Diseases** |  |  |  |  |  |  |  |  |  |  |  |
| Psychiatric Disorders | 8 | 3 (37.5) | 47 (20-74) | 1.5 (0.5-9) | 0/8 | 0/8 | NA | 1.5±0.5 | 492.4±193.3 | 0/4 | 1/6 |
| subacute Stroke | 5 | 3 (60) | 62 (46-86) | 0.5 (<0.5) | 0/5 | 1/5 | NA | 8.6±13.1 | 430.6±169.4 | 0/3 | 2/4 |
| ALS | 15 | 7 (46.7) | 62 (33-86) | 1 (0.5-4) | 0/14 | 0/14 | NA | 2.5±3.0 | 482.1±154.2 | 1/12 | 15/15 |
| PNP | 5 | 2 (40) | 75 (61-80) | 2 (0.5-6) | 2/5 | 0/5 | NA | 1.2±0.5 | 522.0±236.3 | 2/3 | 1/4 |
| SMA | 8 | 4 (50) | 38 (20-50) | 35 (18-47) | 0/6 | NA | NA | 1.0±0 | 298.1±75.0 | NA | 0/1 |
| IIH | 8 | 4 (50) | 26 (18-69) | 0.5 (<0.5-2) | 0/8 | 0/6 | NA | 2.3±1.2 | 539.6±518.7 | NA | 1/3 |
| PAF | 4 | 3 (75) | 76 (73-82) | 4.5 (0.5-9) | 0/4 | 0/4 | 1/1 | 2.8±2.4 | 597.0±161.7 | 0/3 | 1/3 |
| Epilepsy | 7 | 5 (71.4) | 74 (70-92) | 1 (0.5-4) | 0/7 | 0/6 | NA | 2.9±4.5 | 408.3±200.2 | 3/7 | 2/6 |
| Syncope | 2 | 1 (50) | 39 (19-58) | 0.5 (0.5) | 0/2 | 0/1 | NA | 1.0±0 | 462.0±113.1 | 0/1 | 1/1 |
| Others^m^ | 5 | 1 (20) | 63 (31-74) | 2 (0.5-4) | 1/5 | 0/4 | NA | 1.8±0.8 | 558.0±317.9 | 0/2 | 0/1 |

*Abbreviations:* AD, Alzheimer’s Disease; Aβ, Beta-Amyloid; ALS, Amyotrophic Lateral Sclerosis; CAA, cerebral amyloid angiopathy; CSF, cerebrospinal fluid; CT, Computed tomography; DIP, drug-induced Parkinsonism; DLB, dementia with Lewy-Bodies; ET, Essential tremor; FTD, Frontotemporal Dementia; HD, Huntington’s Disease; IIH, Idiopathic intracranial hypertension; iNPH, idiopathic Normal Pressure Hydrocephalus; MCI, Mild Cognitive Impairment; MRI, Magnet Resonance Imaging; MS, Multiple sclerosis; MSA, Multiple System Atrophy, MoCA, Montreal Cognitive Assessment; NA, not available; NfL, Neurofilament light chain; No., Total Number; NPH, Normal Pressure Hydrocephalus; PAF, Pure autonomic Failure; PD, Parkinson’s Disease; PET, Positron Emission Tomography; PNP, Polyneuropathy; PSP, Progressive supranuclear Palsy; pTau181, phosphoTau-181; SMA, Spinal Muscular Atrophy; SPECT, Single Photon Emission Tomography; UD, Unsolved Dementia; UMD, Unsolved Movement Disorder; UPDRS, Movement Disorder Society Unified Parkinson Disease Rating Scale; VP, Vascular Parkinsonism

^a^ median (range) in years, data was available in all patients

^b^ report of cerebral magnet-resonance imaging or computer tomography within last six month describes morphologically aspects of normal pressure hydrocephalus (NPH), number of patients with neuroimaging aspect of NPH/number of patients with available data

^c^ report of magnet resonance imaging within last six month describes suspected diagnosis of cerebral amyloid angiopathy (CAA), number of patients with MRI-suspected CAA/number of patients with available data

^d^ report of Iodine-123-FP-CIT single photon emission tomography or ^18^F-DOPA positron emission tomography within last 24 month describes nigro-striatal degeneration, number of patients with report of nigro-striatal degeneration/number of patients with available data

^e^ Number of white blood cells in CSF/µl, mean±SD, data was available in all patients

^f^ Concentration of total protein in CSF in mg/L, mean±SD, data was available in all patients

^g^ number of patients with evidence of Alzheimer’s Disease biomarkers in CSF (pathological Beta-Amyloid-(1-42)/(1-40)-ratio and/or pathological elevation of phospho-tau181)/ number of patients with available data

^h^ number of patients with reported levels of neurofilament light chain in CSF above 90^th^ percentile corrected for age/ number of patients with available data

^i^ other than MCI-AD

^k^ other than MSA

^m^ Migraine, Myasthenia Gravis, Leukodystrophy, Mitochondriopathy, Traumatic Encephalopathy

| **Discussed diagnosis in**  **unsolved movement disorders (n=12)** | **Neg.**  **αSyn-SAA** | **Pos.**  **αSyn-SAA** |
| --- | --- | --- |
| Lower body parkinsonism (PD or spinal canal stenosis) | 1 | 1 |
| NPH or infantile cerebral palsy | 1 | 0 |
| PD or ET+ | 0 | 1 |
| DIP or NPH | 1 | 0 |
| PD or MSA | 1 | 1 |
| PD or DIP | 2 | 0 |
| FTD-P or paraneoplastic in CUP | 0 | 2 |
| NPH or PSP | 1 | 0 |

**Supplemental Table 2:** Discussed diagnoses with diagnosis “Unsolved Movement Disorder”

*Abbreviations:* αSyn-SAA, alpha-Synuclein Seed Amplification Assay; CUP, cancer of unknown primary; DIP, Drug-induced Parkinsonism; ET+, Essential Tremor-Plus Syndrome; FTD-P, Frontotemporal Dementia-Parkinson; MSA, Multiple System Atrophy; n, total number; Neg., negative; NPH, Normal Pressure Hydrocephalus; PD, Parkinson’s Disease; Pos., positive; PSP, Progressive Supranuclear Palsy;

**Supplemental Table 3:** αSyn-SAA positivity and documented secondary diagnosis

| **Reported Secondary Diagnosis in Patient’s History** | **Neg.**  **αSyn-SAA**  (n=238) | **Pos.**  **αSyn-SAA**  (n=118) | **p-value^a^** |
| --- | --- | --- | --- |
| Polyneuropathy | 22 | 11 | >0.99 |
| Spinal Stenosis | 14 | 10 | 0.34 |
| Stroke | 41 | 22 | 0.77 |
| Arterial Hypertension | 127 | 71 | 0.26 |
| Coronary Heart Disease | 25 | 18 | 0.23 |
| Peripheral Arterial Occlusive Disease | 7 | 4 | 0.76 |
| Current or Past Tobacco use | 16 | 2 | **0.04** |
| Diabetes Mellitus | 36 | 19 | 0.88 |
| Adipositas | 17 | 3 | 0.09 |
| History of Cancer | 35 | 26 | 0.10 |
| Autoimmune Disease | 28 | 8 | 0.44 |
| Renal Failure | 23 | 13 | 0.71 |

^a^ p-value from Fisher’s Exact Test

*Abbreviations:* αSyn-SAA, alpha-Synuclein Seed Amplification Assay; n, number of patients; Neg., negative; Pos., positive

**Supplemental Table 4:** αSyn-SAA positivity and documented CSF parameters

| **CSF parameters** | **Neg.**  **αSyn-SAA** | **Pos.**  **αSyn-SAA** | **p-value^a^** |
| --- | --- | --- | --- |
| Total protein >500mg/L | 100/238 | 52/118 | 0.73 |
| Pleocytosis with ≥5white blood cells/µl | 19/238 | 3/118 | 0.06 |
| Occurrence of oligoclonal bands Type 2 or 3 | 29/173 | 7/89 | 0.06 |
| Disorder of Blood-Brain-Barrier | 57/199 | 21/106 | 0.10 |
| Alzheimer’s Pathology (Aβ-42/40 ratio↓ &/or pTau181↑) | 55/170 | 36/99 | 0.51 |

^a^ p-value from Fisher’s Exact Test

*Abbreviations:* n, number of patients; Neg., negative; Pos., positive; αSyn-SAA, alpha-Synuclein Seed Amplification Assay

**Supplemental Table 5:** Results of αSyn-SAA in patients with selected symptoms

**Supplemental Table 5a:** Reported hyposmia (n=55/210)

|  |  | | | | | | | | | | | | | | | | | sum | |
| --- | --- | --- | --- | --- | --- | --- | --- | --- | --- | --- | --- | --- | --- | --- | --- | --- | --- | --- | --- |
| Pos. SAA No. | 21 | 7 | 3 | 2 | 1 | 1 | 2 | 0 | 0 | 0 | 0 | 0 | 0 | 0 | 0 | 0 | 0 | 37 (98) |  |
| Neg. SAA No. | 0 | 0 | 0 | 0 | 0 | 0 | 2 | 1 | 1 | 1 | 1 | 1 | 1 | 1 | 2 | 3 | 4 | 18 (112) |  |
| Clinical Diagnosis | PD | DLB | AD | PAF | PSP | MCI | UMD | MS | iNPH | FTD | ALS | Encephalitis | MG | PNP | VD | DIP | MSA |  |  |

**Supplemental Table 5b:** Anamnestic suspected Rapid eye movement sleep behavior disorder (n=46/275)

|  |  | | | | | | | | | | sum |
| --- | --- | --- | --- | --- | --- | --- | --- | --- | --- | --- | --- |
| Pos. SAA No. | 15 | 13 | 1 | 1 | 0 | 0 | 0 | 0 | 0 | 0 | 30 (116) |
| Neg. SAA No. | 0 | 0 | 1 | 2 | 1 | 1 | 1 | 1 | 2 | 7 | 16 (159) |
| Clinical Diagnosis | DLB | PD | AD | MCI | UMD | FTD | DIP | Encephalitis | AD-VCI | MSA |  |

**Supplemental Table 5c:** Visual hallucinations in patient’s history (n=40/313)

|  |  | | | | | | | | | | | sum | |
| --- | --- | --- | --- | --- | --- | --- | --- | --- | --- | --- | --- | --- | --- |
| Pos. SAA No. | 19 | 6 | 1 | 3 | 1 | 0 | 0 | 0 | 0 | 0 | 0 | 30 (117) |  |
| Neg. SAA No. | 0 | 0 | 0 | 2 | 1 | 1 | 1 | 1 | 1 | 1 | 2 | 10 (196) |  |
| Clinical Diagnosis | DLB | PD | MCI | AD | AD-VCI | VD | Epilepsy | Stroke | FTD | MSA | UD |  |  |

**Supplemental Table 5d:** Anamnestic suspected orthostatic hypotension (n=45/313)

|  |  | | | | | | | | | | sum |
| --- | --- | --- | --- | --- | --- | --- | --- | --- | --- | --- | --- |
| Pos. SAA No. | 9 | 7 | 4 | 1 | 1 | 0 | 0 | 0 | 0 | 0 | 22 (110) |
| Neg. SAA No. | 0 | 0 | 0 | 15 | 2 | 2 | 1 | 1 | 1 | 1 | 23 (203) |
| Clinical Diagnosis | PD | DLB | PAF | MSA | UMD | AD | VD | UD | PSP | Tumor |  |

**Supplemental Table 5e:** Anamnestic suspected urge incontinence (n=126/353)

|  |  | | | | | | | | | | | | | | | | | | | | | | | | | sum | |
| --- | --- | --- | --- | --- | --- | --- | --- | --- | --- | --- | --- | --- | --- | --- | --- | --- | --- | --- | --- | --- | --- | --- | --- | --- | --- | --- | --- |
| Pos. SAA No. | 20 | 9 | 2 | 7 | 1 | 1 | 2 | 2 | 2 | 2 | 1 | 1 | 0 | 0 | 0 | 0 | 0 | 0 | 0 | 0 | 0 | 0 | 0 | 0 | 50 (118) | |  |
| Neg. SAA No. | 0 | 0 | 0 | 5 | 1 | 1 | 2 | 5 | 6 | 7 | 7 | 16 | 1 | 1 | 1 | 1 | 2 | 2 | 2 | 3 | 3 | 3 | 3 | 4 | 76 (235) | |  |
| Clinical Diagnosis | PD | DLB | PAF | iNPH | VP | ALS | MCI | UMD | AD | AD-VCI | PSP | MSA | Dys | Neuritis | Psy | Encephalitis | UD | PNP | Atx | FTD | MS | VD | Tumor | DIP |  | |  |

**Supplemental Table 5f:** Anamnestic suspected constipation (n=57/353)

|  |  | | | | | | | | | | | | | | | | | | sum |
| --- | --- | --- | --- | --- | --- | --- | --- | --- | --- | --- | --- | --- | --- | --- | --- | --- | --- | --- | --- |
| Pos. SAA No. | 12 | 8 | 1 | 1 | 2 | 2 | 1 | 0 | 0 | 0 | 0 | 0 | 0 | 0 | 0 | 0 | 0 | 0 | 27 (118) |
| Neg. SAA No. | 0 | 0 | 0 | 0 | 2 | 3 | 2 | 1 | 1 | 1 | 1 | 1 | 2 | 1 | 1 | 3 | 3 | 8 | 30 (235) |
| Clinical Diagnosis | PD | DLB | PAF | FTD | AD | UMD | AD-VCI | VD | MCI | DIP | Dys | Epilepsy | SMA | ALS | Psy | PSP | Tumor | MSA |  |

**Supplemental Table 5g:** Anamnestic difficulties falling or staying asleep (n=124/340)

|  |  | | | | | | | | | | | | | | | | | | | | | | | | sum |
| --- | --- | --- | --- | --- | --- | --- | --- | --- | --- | --- | --- | --- | --- | --- | --- | --- | --- | --- | --- | --- | --- | --- | --- | --- | --- |
| Pos. SAA No. | 20 | 13 | 1 | 1 | 1 | 2 | 6 | 3 | 1 | 1 | 0 | 0 | 0 | 0 | 0 | 0 | 0 | 0 | 0 | 0 | 0 | 0 | 0 | 0 | 49 (116) |
| Neg. SAA No. | 0 | 0 | 0 | 1 | 2 | 4 | 12 | 4 | 4 | 7 | 1 | 1 | 2 | 2 | 2 | 2 | 2 | 3 | 3 | 3 | 3 | 4 | 5 | 8 | 75 (224) |
| Clinical Diagnosis | PD | DLB | PAF | VP | iNPH | MCI | AD | UMD | AD-VCI | MSA | Epilepsy | Atx | Encephalitis | MS | DIP | Tumor | ALS | SMA | Dys | VD | UD | Psy | FTD | PSP |  |

**Supplemental Table 5h:**  Depression in patient‘s history (n=126/346)

|  |  | | | | | | | | | | | | | | | | | | | | | | | | | | sum |
| --- | --- | --- | --- | --- | --- | --- | --- | --- | --- | --- | --- | --- | --- | --- | --- | --- | --- | --- | --- | --- | --- | --- | --- | --- | --- | --- | --- |
| Pos. SAA No. | 13 | 13 | 2 | 7 | 3 | 1 | 1 | 1 | 1 | 0 | 0 | 0 | 0 | 0 | 0 | 0 | 0 | 0 | 0 | 0 | 0 | 0 | 0 | 0 | 0 | 0 | 42 (118) |
| Neg. SAA No. | 0 | 0 | 0 | 8 | 5 | 2 | 3 | 4 | 7 | 1 | 1 | 1 | 1 | 2 | 2 | 2 | 2 | 2 | 2 | 4 | 4 | 4 | 5 | 6 | 7 | 9 | 84 (228) |
| Clinical Diagnosis | PD | DLB | VP | AD | UMD | AD-VCI | iNPH | FTD | MCI | Leuk | IIH | Atx | SMA | Encephalitis | Dys | Chorea | Tumor | PNP | ALS | PSP | DIP | UD | MS | Psy | VD | MSA |  |

***Supplemental Table 5a-h*** *Abbreviations:* AD, Alzheimer’s Disease; ALS, Amyotrophic Lateral Sclerosis; Atx, Ataxia; DIP, drug-induced Parkinsonism; DLB, dementia with Lewy-Bodies; Dys, Dystonia; FTD, Frontotemporal Dementia; IIH, Idiopathic intracranial hypertension; iNPH, idiopathic Normal Pressure Hydrocephalus; Leuk, Leukodystrophy, MCI, Mild Cognitive Impairment other than AD; MG, Myasthenia Gravis; MS, Multiple sclerosis; MSA, Multiple System Atrophy, Neg., negative; No., Total number of patients; PAF, Pure autonomic Failure; PD, Parkinson’s Disease; PNP, Polyneuropathy; Pos., positive; PSP, Progressive Supranuclear Palsy; Psy, Psychiatric diagnosis; SAA, Seed Amplification Assay; SMA, Spinal Muscular Atrophy; UD, Unsolved Dementia; UMD, Unsolved Movement Disorder; VCI Vascular Cognitive Impairment; VP, Vascular Parkinsonism
